# The Influence of Mixing Methods of Epoxy Composition Ingredients on Selected Mechanical Properties of Modified Epoxy Construction Materials

**DOI:** 10.3390/ma14020411

**Published:** 2021-01-15

**Authors:** Izabela Miturska, Anna Rudawska, Miroslav Müller, Monika Hromasová

**Affiliations:** 1 Department of Production Engineering, Faculty of Mechanical Engineering, Lublin University of Technology, Nadbystrzycka 36, 20-618 Lublin, Poland; a.rudawska@pollub.pl; 2 Department of Material Science and Manufacturing Technology, Faculty of Engineering, Czech University of Life Sciences, Kamýcká 129, 165 21 Prague, Czech Republic; muller@tf.czu.cz; 3 Department of Electrical Engineering and Automation, Faculty of Engineering, Czech University of Life Sciences, Kamýcká 129, 165 21 Prague, Czech Republic; hromasova@tf.czu.cz

**Keywords:** epoxy construction materials, physical modification, mechanical properties

## Abstract

The proper process of preparing an adhesive composition has a significant impact on the degree of dispersion of the composition ingredients in the matrix, as well as on the degree of aeration of the resulting composition, which in turn directly affects the strength and functional properties of the obtained adhesive compositions. The paper presents the results of tensile strength tests and SEM microphotographs of the adhesive composition of Epidian 57 epoxy resin with Z-1 curing agent, which was modified using three fillers NanoBent ZR2 montmorillonite, CaCO_3_ calcium carbonate and CWZ-22 active carbon. For comparison purposes, samples made of unmodified composition were also tested. The compositions were prepared with the use of six mixing methods, with variable parameters such as type of mixer arm, deaeration and epoxy resin temperature. Then, three mixing speeds were applied: 460, 1170 and 2500 rpm. The analyses of the obtained results showed that the most effective tensile results were obtained in the case of mixing with the use of a dispersing disc mixer with preliminary heating of the epoxy resin to 50 °C and deaeration of the composition during mixing. The highest tensile strength of adhesive compositions was obtained at the highest mixing speed; however, the best repeatability of the results was observed at 1170 rpm mixing speed. Based on a comparison test of average values, it was observed that, in case of modified compositions, the values of average tensile strength obtained at mixing speeds at 1170 and 2500 rpm do not differ significantly with the assumed level of significance *α* = 0.05.

## 1. Introduction

The strength of structural adhesive joints depends on many technological and constructional factors. The most important factors, the optimization of which may significantly increase the strength of structural adhesive joints, include the method of preparing the surfaces of the bonded elements, the type of adhesive used and the method of its preparation, the manufacturing technology considering the geometry of the joint, the method of loading and the conditions of curing the adhesive joint [1,2].

The procedure of the composition preparation, therefore, has a direct impact on the strength properties of the created joints. The correct process of preparation of the adhesive composition has a significant impact on the degree of dispersion of the composition ingredients in the matrix, as well as on the degree of aeration of the created composition. Therefore, the strength and functional properties of the final material depend on these parameters. It should be emphasized that the mixing process depends on many factors, including the type and properties of the components (including viscosity, chemical composition), the amount of the mixture prepared (which is of great importance due to the exothermic reaction) and also the order of adding the ingredients. The mixing method and the technological parameters of this process are very important, as it determines, e.g., the correct mixing of the components (no agglomerates in the adhesive composition, no sedimentation of the components, no gas bubbles, etc.). For this reason, it is very important to develop a technology for mixing the components of adhesive compositions.

The main type of adhesives used in the process of structural bonding of metals and composite materials are epoxy adhesives. The versatility of epoxy adhesives results from a large number of combinations of epoxy resins and curing agents, with a different chemical composition and different curing methods, which results in different molecular structure of the obtained polymer [3,4]. Continuous development of modern constructions forces these compositions to be modified to improve their properties.

By reason of these aspects, numerous experimental, often destructive, experiments are conducted, which take up the subject of testing the strength of epoxy adhesive compositions in the cured state. Due to the increasing demands on the strength of adhesive bonds, an important direction of research is to subject adhesive compositions to modifications.

There are three types of modifications distinguished in the literature [4,5,6]:

- Chemical;
- Physical;
- Physic-chemical (combining the two mentioned above).

Physical modification occurs by physical effects, including mixing. Modified polymers (including adhesives) differ from those before the modification in the structure, physical, functional, and visual properties. The most common methods of physical modification are the addition of fillers. The operational properties of the modified materials significantly depend on the type of the filler used (particle shape and size, specific surface, concentration of dispersed phase) [7,8]. The best properties are obtained by introducing the smallest possible fillers, preferably with particle sizes measured on a nanometric scale [9,10]. It should be noted that smaller particles (e.g., nano-scale) disperse better in the resin matrix, hence a more homogeneous structure of the resulting compositions is obtained. Even a small modifying additive may improve certain characteristics of adhesive materials.

Primary particles of the adhesive filler in the non-agglomerated form are extremely rare in real conditions. Because of their binding by forces of physicochemical interaction, in the number of a few or more primary particles, an aggregate is formed. A cluster of several or more aggregates bound by forces of physical or chemical interaction forms an agglomerate. The occurrence of nanoparticles agglomerates in the matrix may cause the formation of defects, which results in the reduction in the properties of the modified polymer; therefore, it is important to select an appropriate method of mixing the adhesive composition, which will enable to obtain the required dispersion of nanoparticles and their proper wetting by the matrix material [5,10,11,12,13]. This aspect is important at the stage of preparation of the adhesive composition, then its application, and may significantly affect the properties of the adhesive joint.

The preparation of epoxy adhesive containing filler particles can be performed by direct mixing. It consists in direct introduction of the filler into the liquid resin and mixing the components in a mechanical or ultrasonic way. Mechanical mixing can be carried out, for example, by means of a high-speed mixer. Mechanical mixing can be carried out using laboratory and technical mixers. Mixers designed for research purposes are usually laboratory mixers. They are used for mixing resins with various inorganic and organic fillers. Depending on the manufacturer and type of mixer, the capacity of the mixing container can range from 1 to 70 L, and the speed of the mixing tool can be from several hundred to several tens of thousands rpm. The diameter of the mixer depends on the size of the container. Another way of dispersing the filler in the epoxy adhesive is ultrasonic mixing. It consists in applying alternating pressure caused by an acoustic wave in the area above the cavitation formation threshold in the solution. Ultrasonic mixing can be carried out using ultrasonic homogenizers, which can be used as laboratory or technical equipment. They are equipped with probes from 3 to 40 mm in diameter, thus allowing homogenization of samples from 5 to 2000 mL.

A typical course of the process of preparing unmodified adhesive compositions using the mechanical mixing method, which is recommended by adhesive manufacturers, can be carried out according to the following methodology:

- Epoxy resin application, at room temperature directly before application;
- Introduction of a sufficient amount of hardener into the resin;
- Mechanical mixing of adhesive ingredients for 2 min.

The influence of the adhesive mixing method on the strength of adhesive joints was described by authors of numerous publications [10,14,15].

The paper presents comparative results of tensile strength tests of an adhesive composition modified with three fillers: NanoBent ZR2 montmorillonite, CaCO_3_ calcium carbonate and CWZ-22 active carbon. Epoxy adhesive compositions were prepared using 6 mixing methods, at variable mixing speeds. The obtained results were subjected to precise statistical analysis. The presented results of statistical analyses were conducted using the Statistica program. SEM images were also taken to compare the dispersion of fillers in the compositions depending on the speed during mixing of adhesive ingredients.

## 2. Materials and Methods

The aim of the study was to prepare and determine the strength properties of compositions of epoxy adhesives modified with natural fillers.

### 2.1. Ingredients of Adhesive Compositions

The studies used adhesive compositions based on Epidian 57 epoxy resin and Z-1 curing agent.

Epidian 57 (producer: CIECH Sarzyna, Nowa Sarzyna, Poland) is a mixture of epoxy resin made of bisphenol A and epichlorohydrin with an average molecular weight ≤700 and thinner (saturated polyester resin). Its main application is in adhesives for bonding metals, glass, ceramics, and wood [6,16]. Epidian 57 has an epoxy number ≥0.40 mol/100 g, viscosity (at 25 °C): 13,000–19,000 mPa s and density (at 20 °C): 1.14–1.17 g/cm^3^ [16,17,18].

Z-1 curing agent is an aliphatic amine, which is used mainly in compositions with low-molecular epoxy resins and products based on them. When the curing process begins, a certain amount of time is left to use the mixture before curing (open time). The gelation time is about 35 min at room temperature. Initial curing is achieved after 48 h—the degree of curing is about 80–90%, and the whole-white curing is after 7–14 days [19]. This hardener is characterized by viscosity (at 25 °C): 20–30 mPa s, density (at 20 °C): 0.978–0.983 g/cm^3^ and amine number min. 1100 mg KOH/g [10,19].

### 2.2. The Fillers Used in the Studies

The adhesive compositions were modified with three fillers: NanoBent ZR2 montmorillonite, CaCO_3_ calcium carbonate and CWZ-22 active carbon.

Montmorillonite is a filler with a high degree of crushing (i.e., a filler with micro and nano particle size) under the trade name NanoBent ZR2 (producer: ZGM “Zębiec” S.A. in Zębiec, Poland). NanoBent ZR2 is an aluminosilicate modified with quaternary ammonium salt. NanoBent ZR2 montmorillonite can be used as a double action: thixotropic and biocidal additive.

CaCO_3_ calcium carbonate in the powder form (producer: ZPW Trzuskawica S.A. in Siatkówka, Poland) was also used in the research. Its molecular weight is 100.09 g/mol. Typical concentration of CaCO_3_ is 98.23%, while the concentration range is 92–99% [20].

The third filler used during the research was CWZ-22 active carbon in the dusty form (producer: Stanlab sp. z o.o., Lublin, Poland), with a molar mass of 12.01 g/mol. The production of active coals is based on natural organic raw materials of polymeric structure. Wood (35% of the total raw material consumption), hard coal (28%), lignite (14%), peat (10%) and, locally, also waste products, nut shells or fruit stones (10%) are used for this purpose. Because of its properties and affordability, active carbon is used in medicine and cosmetics (as medicinal charcoal and a component of cosmetic product formulas), in the chemical industry (as a catalyst and solid carrier for other catalysts), in technology (as a component of gas scavengers, e.g., in gas masks and protective clothing), in electrical engineering (as a material allowing to obtain large capacities in super-condensers) or in water treatment. The presence of carbon as a powder filler in a hardened polymer matrix may significantly change not only its thermal properties but also its strength. Mechanical properties are the most interesting by reason of their constructional features. Knowledge of these properties, determined by the action of forces of appropriate distribution and size, would allow us to roughly predict the behavior of the composite material under real working conditions.

The chemical formulas for the materials used in the tests are presented in Table 1.

The determination of the composition and the amount of ingredients subjected to the tests is shown in Table 2.

The quantities of fillers were selected on the basis of our own experimental research and literature review [21,22,23,24,25,26,27,28].

### 2.3. Preparation of Adhesive Compositions

During the preparation of samples for strength tests, mechanical mixing was used in the process of mixing the adhesive compositions.

**Figure 1:**
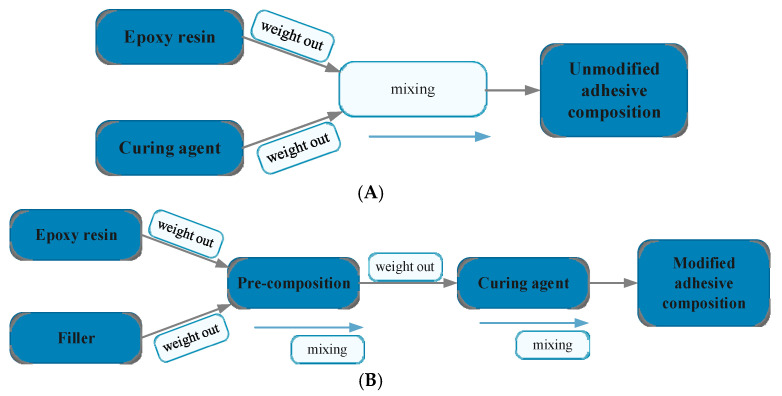
Schematic diagram of the sequence of activities performed during preparation: (**A**) unmodified adhesive compositions and (**B**) modified adhesive compositions.

The sequence of the stages of preparation of the adhesive compositions was carried out according to the scheme presented in Figure 1.

At the beginning, a typical procedure of the preparation process of the adhesive compositions mechanical mixing (which in the further part of the work was marked as a variant of mixing 1) was applied, which took place according to the following methodology:

- Weighing out a specific amount of epoxy resin, which was stored at room temperature directly before application;
- Introducing a filler into the epoxy resin (for modified compositions) in an appropriate amount;
- Mechanical mixing of composition ingredients for 3 min at 460 rpm;
- Introducing into the epoxy resin mixed with filler curing agent in appropriate amounts;
- Mechanical mixing of the adhesive ingredients at a mixing speed—460 rpm for 2 min.

In case of unmodified compositions, the stage of introducing the filler into the epoxy resin was omitted. Further variants of mixing were also performed at the mixing speed—460 rpm, with the use of variable parameters, which were: the geometry of the mixer, mixing speed, additional venting process and heating of the epoxy resin. The parameters characterizing individual mixing processes are presented in Table 3.

A descriptive overview of the different methods is presented in Table 4.

By selecting the most effective mixing method, the influence of the mixing speed of adhesive composition ingredients on their strength properties was also tested. Apart from the basic mixing speeds at 460, 1170 and 2500 rpm were used.

**Figure 2:**
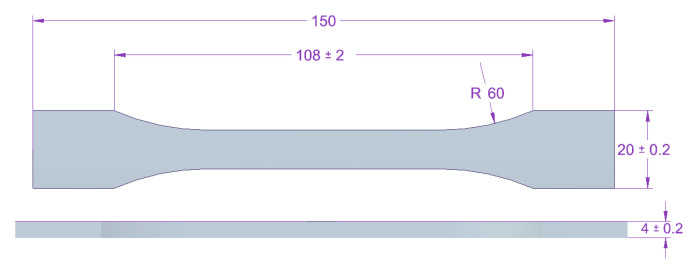
Shape and dimensions of adhesive compositions sample for tensile strength testing according to PN EN ISO 527-2 [29]. (Unit: mm)

Adhesive composition samples in the cured state were tested. For tensile strength tests, dumb-bell type 1B samples were used, according to PN EN ISO 527-2 standard. The dimensions of the used samples are shown in Figure 2.

To meet each of the parameters characterizing the different mixing methods, it was necessary to use appropriate stands and tools. Weighing of the ingredients of adhesive compositions was performed using KERN CKE 3600-2 laboratory balance with the measurement accuracy of ±0.01 g. Mixing of the compositions was performed using two types of mixers, differing in geometry. The first one was a paddle mixer.

The second type of mixer was a dispersing disc mixer with holes and trapezoidal teeth.

The mixers were made based on the applicable standards [30,31,32], so that the dimensions of the mixers were appropriate in relation to the vessel in which the adhesive compositions were mixed.

Deaeration of the compositions during their mixing was performed with the use of a two-stage vacuum pump model VP6D. (CPS, Mareza, Poland)

Mixing was carried out by adapting OPTIMUM B20 (Optimum Maschinen, Hallstadt, Germany) and Güde GTB 16/5 A (Güde, Wolpertshausen, Germany) table-top drilling machine, which enabled the adjustment of the mixer’s rotation speed.

The epoxy resin heating stage was carried out using an electric heater (DEPILUX 400) (Activ, Wroclaw, Poland) with the power of 100 W, which allows for smooth regulation of the liquid heating from 45 to 105 °C. The temperature of the heated epoxy resin was monitored using an electronic thermometer (Amarell Electronic, Kreuzwertheim, Germany) with the measuring range of −50 to 200 °C and the measuring accuracy of ±0.1 °C.

The samples of epoxy adhesive compositions were prepared at the temperature of 23 ± 2 °C with air humidity of 23 ± 3% and then subjected to a one-stage cold curing process for 7 days in unchanging conditions. The technological conditions for mixing and curing process have been selected on the basis of manufacturers’ guidelines and research literature [14,33]. For each composition and for each mixing method 10 dumb-bell specimen of adhesive in the cured state were made. Ten dumb-bell specimens from each material were produced by casting mixed compositions in single dumb-bell-shaped molds.

### 2.4. SEM Analysis

The results of scanning electron microscopy (SEM) analysis were performed on the Tescan MIRA3 microscope (Tescan Orsay Holding, Brno—Kohoutovice, Czech Republic), and they complement the mechanical tests results. The results of a fracture surface help clarify the changes in mechanical properties presented in graphs. The prepared samples were dusted with 5 µm of gold. The parameters of SEM samples are evident in a bottom description part of figures, i.e., enlargement, HV in kV, type of detector SE (secondary electrons) and BSE (backscattered electrons).

### 2.5. Statistical Analysis

Analyzing the results of the experimental studies, descriptive statistics have been determined, which makes it possible to determine the characteristics of the feature being tested. Position measures and scattering measures were determined. The position measures are used to determine the value of the variable described by the distribution around which all other values of the variable are concentrated. Among the position measures, an average value and a median were determined. The scattering measures are used to examine the degree of variation of the variable value. Among the measures of scattering, a quartile range was determined.

The next step of statistical analysis was to carry out the uniformity of variance test. For this purpose, the Levene test was used to test the homogeneity of variance of two or more variables or groups.

The normality of the distribution and the assumption of homogeneity of the variance were checked in order to select the test for calculating further statistics:

- If the data in question do not meet the uniformity of variance assumption and do not have a normal distribution, non-parametric tests should be used.
- If the data under consideration meet the assumptions of uniformity of variance and have a normal distribution, parametric tests should be used.

When non-parametric tests are required, the Kruskal–Wallis test was used to compare several averages from many populations or multiple samples.

If the conditions for the use of parametric tests were met, then ANOVA analysis was used. This is the analysis to compare the averages of several groups in order to clarify the differences detected by the analysis of variance, so that they can group the averages and extract homogeneous groups, i.e., those which do not differ statistically from each other.

Statistical analyses presented in the paper were conducted in the Statistica 13 program.

## 3. Results

After the curing period, the adhesive compositions were subjected to strength and microscope tests. Tensile strength was analyzed in the study. In general, strength means resistance to external factors, in this case, tensile (destructive) force, which is very important from the point of view of designing adhesive joints [1].

### 3.1. Strength Tests

**Figure 3:**
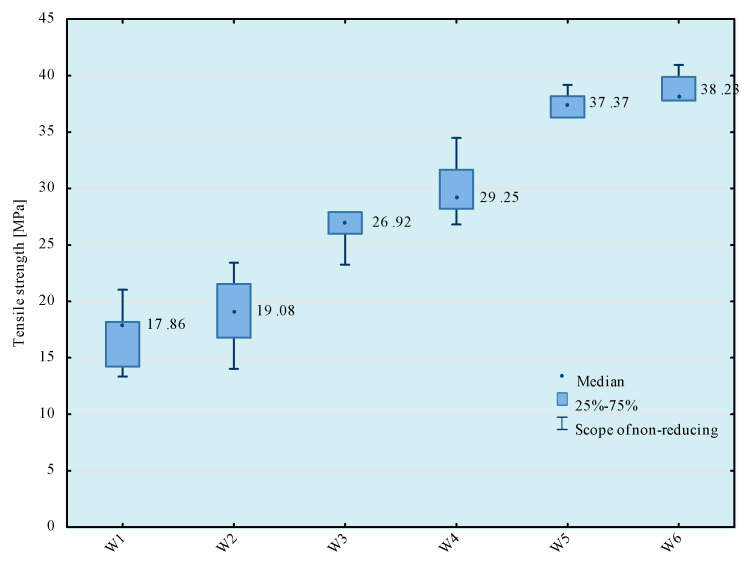
Tensile strength of E57/Z-1/100:10 composition depending on the mixing method.

**Figure 4:**
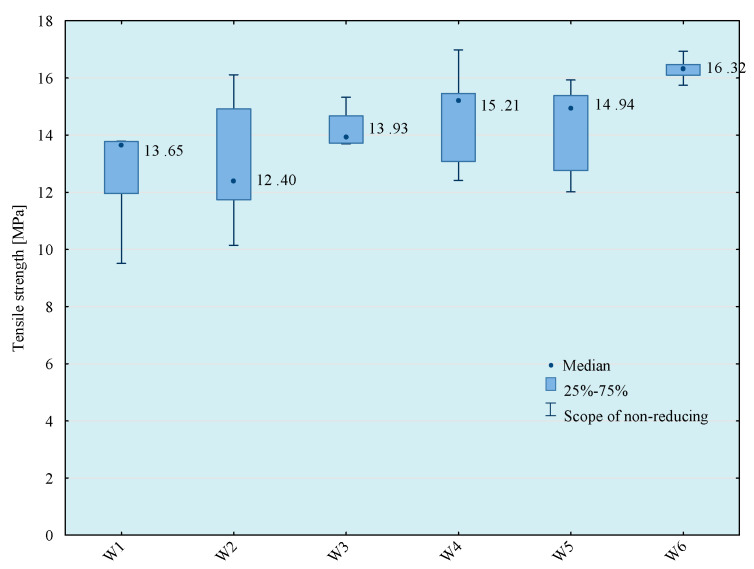
Tensile strength of E57/Z-1/ZR2/100:10:5 composition depending on the mixing method.

**Figure 5:**
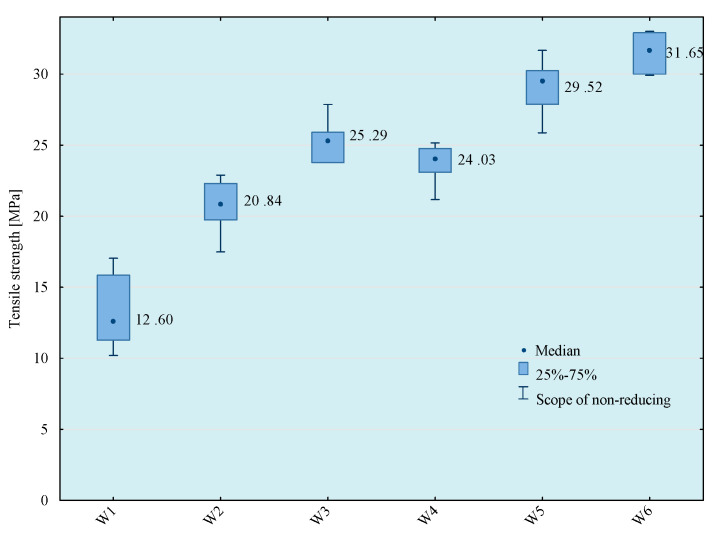
Tensile strength of E57/Z-1/CaCO_3_/100:10:20 composition depending on the mixing method.

**Figure 6:**
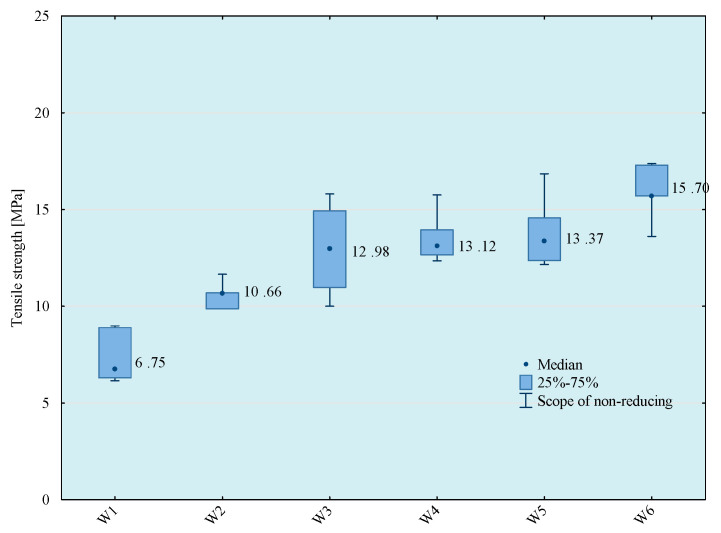
Tensile strength of E57/Z-1/CWZ-22/100:10:20 composition depending on the mixing method.

Tests of tensile strength of adhesive compositions were carried out on the Zwick Roell Z150 (Zwick/Roell, Wroclaw, Poland) strength machine, according to the PN EN ISO 527-1 standard [29]. The crosshead speed during the test was 5 mm/min. The initial tensile force was 30 N. The obtained results of the strength tests are presented in Figure 3, Figure 4, Figure 5 and Figure 6. The median value and quartile spacing, which illustrate the range of obtained tensile strength values, are presented.

Then, the normal distribution of the results obtained was checked using the W Shapiro–Wilk test. The results of the test are shown in the table at the end of the paper as Table A1.

From the results obtained, for the Shapiro–Wilk test, the p level in all groups is greater than the assumed significance level *α* = 0.05, and therefore, the distribution should be assumed to be in accordance with normal distribution. The assumption of equal variance was then analyzed using the Levene test. The results are shown in the table at the end of the paper as Table A2.

The Levene test shows that the assumption of uniformity of variance is met (*p* > 0.05). ANOVA variance analysis was then performed using a post hoc test. The results of Tukey’s test (HSD) are presented in the table at the end of the paper as Table A3, Table A4, Table A5 and Table A6.

The presented analyses show that the most effective results of tensile strength of adhesive compositions were obtained using the 5th and the 6th mixing methods, because the average tensile strength values for these mixing methods are in the same homogeneous groups. Therefore, in further analyses, the 5th mixing method was applied, which, in comparison with the 6th mixing method, shortens the time of preparation of the adhesive composition by the time allocated for bleeding the composition after the mixing process.

**Figure 7:**
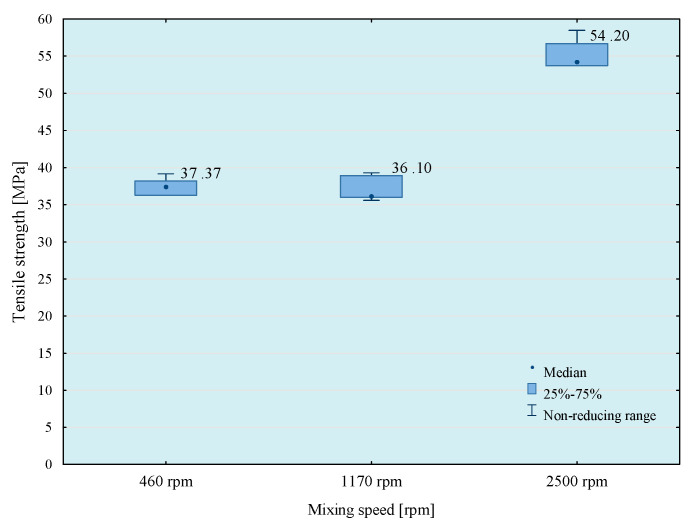
Tensile strength of E57/Z-1/100:10 composition as a function of mixing speed.

**Figure 8:**
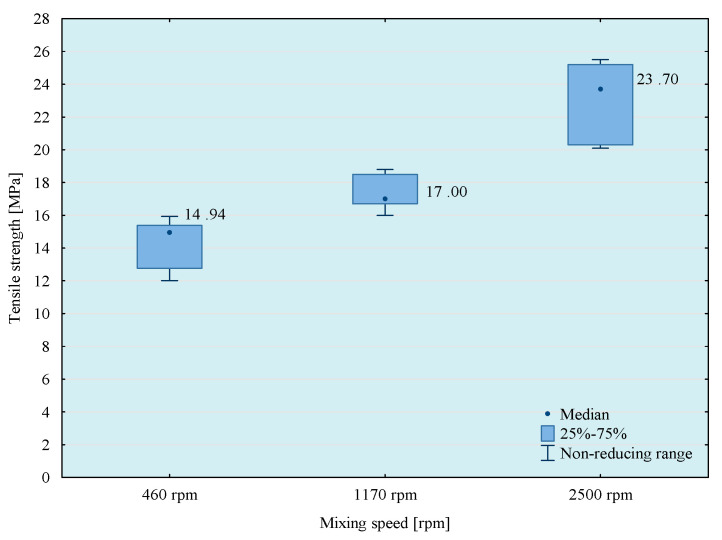
Tensile strength of E57/Z-1/ZR2/100:10:5 composition as a function of mixing speed.

**Figure 9:**
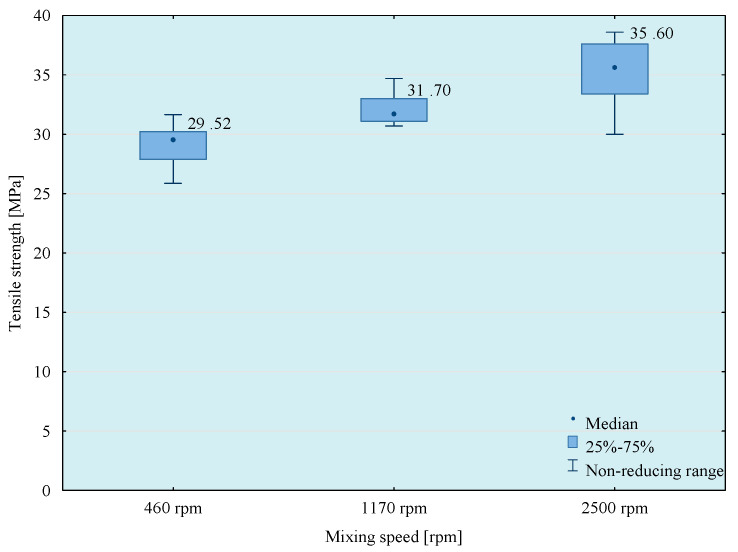
Tensile strength of E57/Z-1/CaCO_3_/100:10:20 composition as a function of mixing speed.

**Figure 10:**
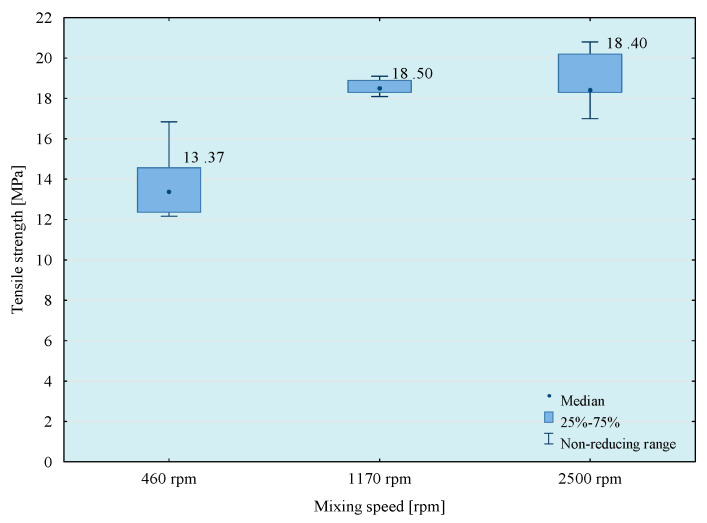
Tensile strength of E57/Z-1/CWZ-22/100:10:20 composition as a function of mixing speed.

In the next stage of research, the influence of mixing speed of the composition on its strength properties was checked. For this purpose, compositions were prepared using the 5th mixing methods. The results of the conducted tests are presented in Figure 7, Figure 8, Figure 9 and Figure 10.

The normal distribution of the results obtained was then checked. The results of the conducted test are presented in Table 5.

Based on the results obtained, it can be assumed that the distribution is consistent with normal distribution. The assumption of equal variance was then analyzed. The results are presented in Table 6.

The Levene test shows that the assumption of homogeneity of the variance in one group has not been met. The non-parametric Kruskal–Wallis test was then applied, the results of which are summarized in Table 7.

From the results obtained, the highest strength was obtained when mixing at the highest speed, but the best repeatability of the results was observed when using 1170 rpm mixing speed. Based on a comparison test of average values, it can be observed that in the case of modified compositions, the values of average tensile strength obtained at the mixing speeds—1170 and 2500 rpm do not differ significantly with the assumed level of materiality *α* = 0.05. It should be noted, however, that mixing of adhesive compositions with mixing speed at 2500 rpm in a glass container is slightly dangerous. Based on the obtained conclusions and observations, the mixing speed at 1170 rpm was used in further studies. However, to comprehensively assess the influence of the mixing speed on the properties of the tested compositions, it was necessary to perform a microscopic analysis of the structure of these compositions. Therefore, SEM photos of samples prepared by five methods of mixing with variable mixing speeds were taken. Failure of the samples was examined and then sprayed with gold using Quorum Q150R ES (Quorum, Laughton, UK)—spreading deposition rate.

### 3.2. SEM Analysis

It follows from the research results of Fu et al. that mechanical properties of particle composites depend on a suitable choice of a filler, on an interfacial interaction between a matrix and the filler [34], on the size of the used particles, on their distribution in the composite system and on their concentration of course.

**Figure 11:**
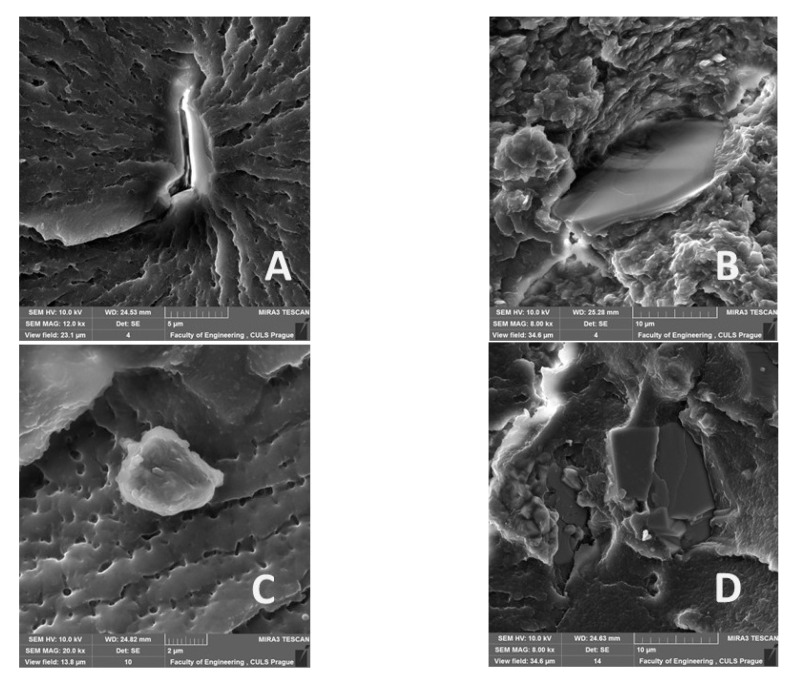
SEM images of fracture surface: (**A**) epoxy resin—mixing speed—460 rpm (MAX 12.00 k×, (**B**) composite material with filler NanoBent ZR2 montmorillonite—mixing speed—1700 rpm (MAG 8.00 k×), (**C**) composite material with filler CWZ-22 active carbon—mixing speed—460 rpm (MAG 20.00 k×), (**D**) composite material with filler CaCO_3_ calcium carbonate—mixing speed—460 rpm (MAG 8.00 k×).

**Figure 12:**
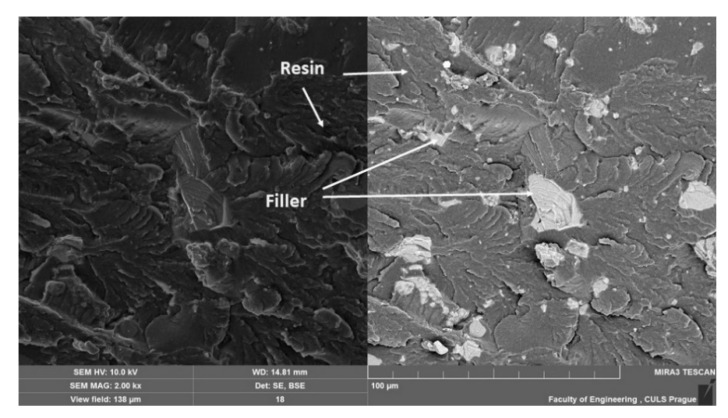
SEM images of fracture surface with filler CaCO_3_ calcium carbonate, mixing speed—460 rpm (MAG 2.00 k×). SE figure is shown on the left and the BSE on the right.

**Figure 13:**
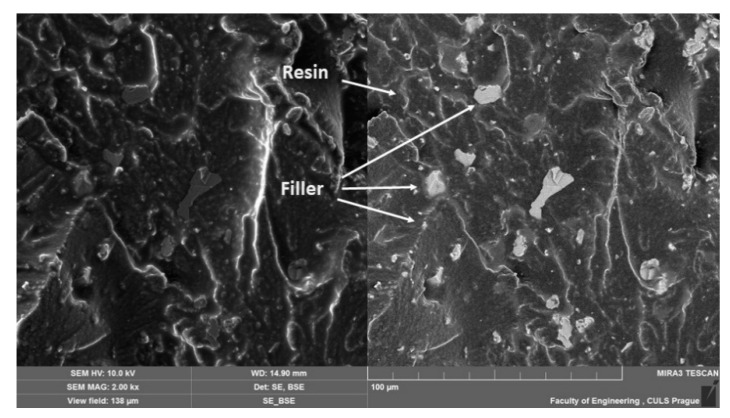
SEM images of fracture surface with filler CaCO_3_ calcium carbonate, mixing speed—1170 rpm (MAG 2.00 k×). SE figure is shown on the left and the BSE on the right.

**Figure 14:**
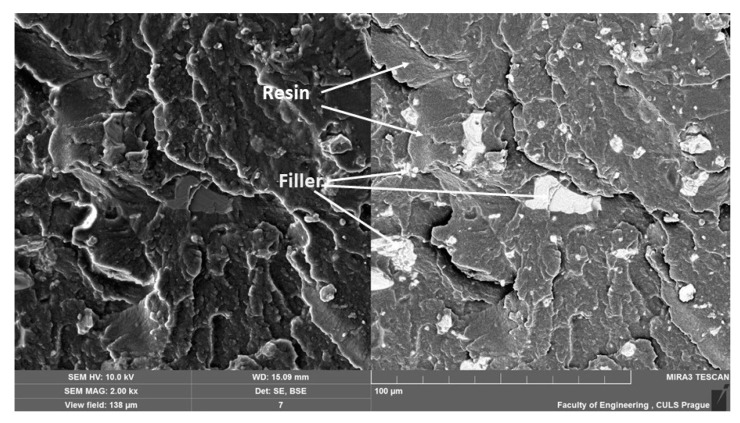
SEM images of fracture surface with filler CaCO_3_ calcium carbonate, mixing speed—2500 rpm (MAG 2.00 k×). SE figure is shown on the left and the BSE on the right.

**Figure 15:**
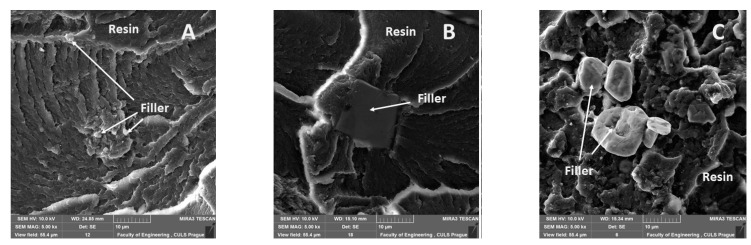
SEM images of fracture surface with filler montmorillonite (NanoBent ZR2), (MAG 5.00 k×): (**A**) mixing speed—460 rpm, (**B**) mixing speed—1700 rpm, (**C**) mixing speed—2500 rpm.

**Figure 16:**
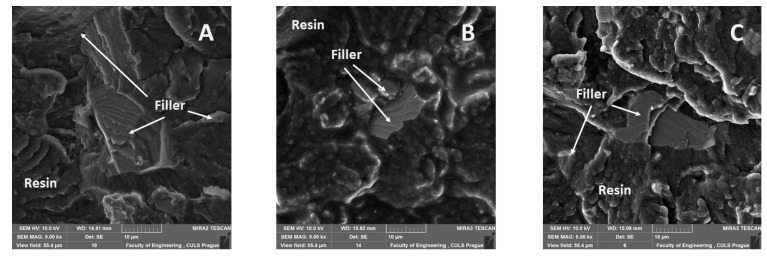
SEM images of fracture surface with filler CaCO_3_ calcium carbonate (MAG 5.00 k×): (**A**) mixing speed—460 rpm, (**B**) mixing speed—1700 rpm, (**C**) mixing speed—2500 rpm.

**Figure 17:**
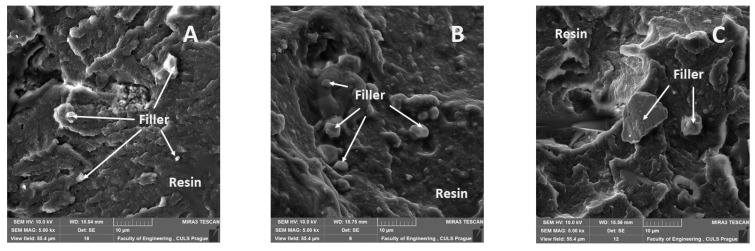
SEM images of fracture surface with filler CWZ-22 active carbon (MAG 5.00 k×): (**A**) mixing speed—460 rpm, (**B**) mixing speed 1700—rpm, (**C**) mixing speed—2500 rpm.

Figure 11, Figure 12, Figure 13, Figure 14, Figure 15, Figure 16 and Figure 17 presents the research results of fracture surfaces of individual tested variants, i.e., an influence of mixing speeds at 460, 1170 and 2500 rpm of unmodified adhesive composition and composites with the filler NanoBent ZR2 montmorillonite, CaCO_3_ calcium carbonate and CWZ-22 active carbon.

Figure 11A presents a detailed view on the fracture surface after a static tensile test. A rise in cracks, which were in micrometers, occurred at a smaller mixing speed during a hardening process. The interaction between the epoxy resin and the filler is evident from Figure 11B–D.

An overall picture of the fracture surface and filler distribution in the composite structure of the tested materials is evident from Figure 12, Figure 13 and Figure 14, which present the fracture surface of the composite material with the filler CaCO_3_ calcium carbonate. SE and BSE detectors were used for the research on the filler distribution in the composite structure at SEM analysis. Based on the SEM images, it can be seen that the better distribution of the calcium carbonate CaCO_3_ filler occurred at a higher speed during mixing. The calcium carbonate particles are more dispersed in the epoxy resin matrix, which is noticeable in Figure 12. SEM images presented in Figure 13, Figure 14 and Figure 15 with the filler CaCO_3_ calcium carbonate are stated as an example. A huge dimensional variability of the filler is also noticeable. Wettability of the filler and the epoxy resin did not influence mixing speeds of the mixture, i.e., 460, 1170 and 2500 rpm.

The interaction between various fillers NanoBent ZR2 montmorillonite, CaCO_3_ calcium carbonate, CWZ-22 active carbon and the epoxy resin at individual tested mixing speeds at 460, 1170 and 2500 rpm are evident from Figure 15, Figure 16 and Figure 17.

A good wettability of the surface between the matrix (epoxy resin) and the filler, which is a basic presumption for a successful production of composite materials, is visible at the same enlargement of the samples presented in Figure 15, Figure 16 and Figure 17, i.e., MAG 5.00 k×. The good wettability in the interface of the filler and the epoxy resin increases the transfer of a stress inside the composite layer. The matrix (epoxy resin) based on thermoset polymer created a brittle fracture, which is evident, e.g., from Figure 15A.

## 4. Discussion

**Figure 18:**
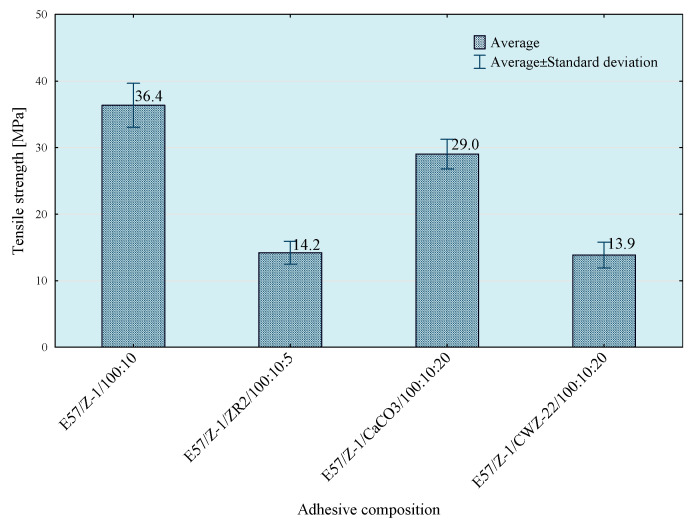
Results of tests of average tensile strength of compositions prepared with 5 mixing methods at 460 rpm.

**Figure 19:**
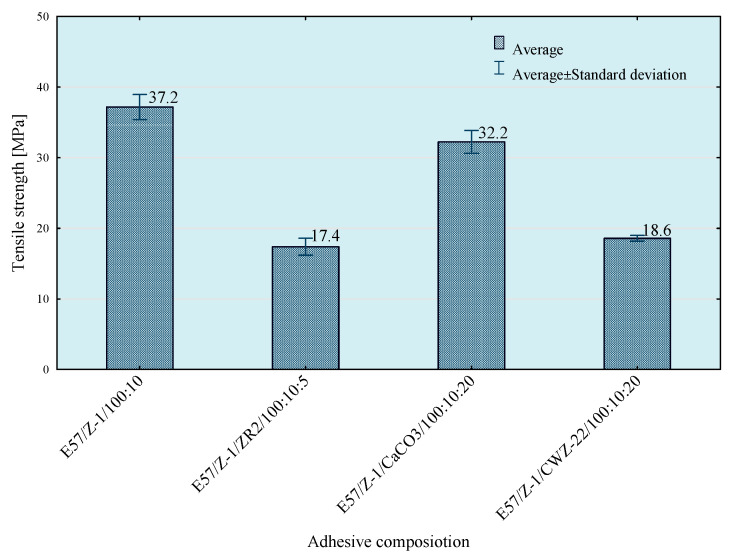
Results of tests of average tensile strength of compositions prepared with 5 mixing methods at 1170 rpm.

**Figure 20:**
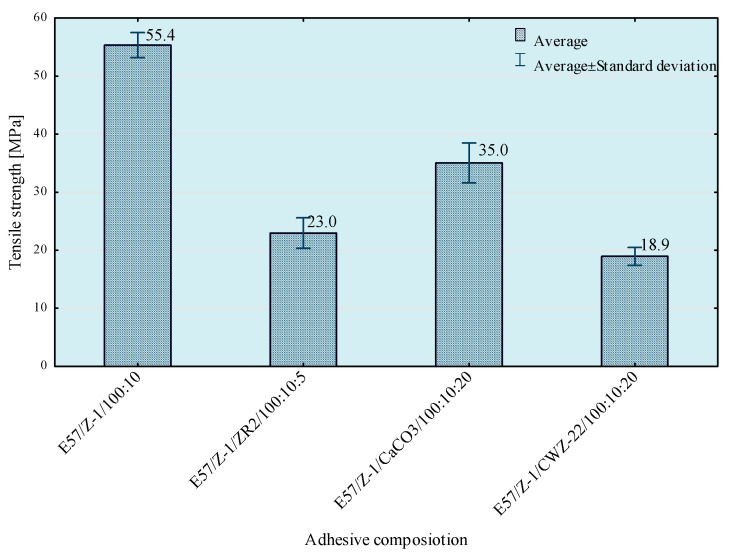
Results of tests of average tensile strength of compositions prepared with 5 mixing methods at 2500 rpm.

To enable a comprehensive comparison of the influence of the mixing speed of the composition ingredients on its properties, the average values of tensile strength for individual compositions and mixing speed are presented in Figure 18, Figure 19 and Figure 20.

Based on the results presented above, it can be observed that the highest tensile strength values were obtained for reference compositions. In case of the lowest mixing speed, the disproportions between the results for particular compositions are smaller than in the case of compositions prepared in the process of mixing with higher speeds. This is because in the case of mixing at higher speeds higher strength values are obtained, which may be the result of better dispersion and the degree of mixing of composition ingredients.

According to the authors of the publication [14,15], the influence of the mixing process has a significant impact on the properties of structural adhesives. In the opinion of Michels et al. [14], an initial epoxy exposure to high temperature accelerates the curing and allows for a much faster strength and stiffness development. In the presented study results, it was also observed that preheating of the epoxy resin improved tensile strength on average by 15%. According to Halder et al. [15], a proper selection of the mixing method improves the quality of the dispersion of filler particles introduced into the adhesive composition matrix, which in turn improves tensile strength.

The presented results and SEM photos showed that increasing the mixing speed resulted in the improvement in the dispersion of particles in the structure of the obtained compositions and, consequently, increased the value of tensile strength by about 30% on average. The presented SEM results indicated high dispersion of filler particles in the epoxy resin matrix. In the papers [11,12,13], the authors emphasized that wettability of filler surfaces has an important role. In addition, the SEM analysis also showed a reduction in porosity in the structure of the samples of adhesive compositions, during preparation of which a venting process was applied during mixing. Bittmann et al. [11] underlined that in order that the conventional microscale fillers and nanofillers surface can interact with the matrix material, a good dispersion, i.e., a good separation and a homogeneous distribution of the nanoparticles into the polymer, is required. In this work, ultrasonic waves generated by an ultrasonic horn were used to disperse titanium dioxide nanoparticles into epoxy resin. Matykiewicz [12] presented the review of various studies into the mechanical and thermo mechanical properties of hybrid epoxy composites with both powder and fiber filler. The significant role of not only the type of fillers, but also the modification of the epoxy matrix was emphasized in order to ensure good adhesion between all components in the laminate to provide the increased mechanical and thermomechanical properties of the hybrid composite. This was shown in the SEM images in [12].

However, it should be stressed that the indicated values are important for the epoxy compositions tested in this study.

## 5. Conclusions

The carried-out research was mainly aimed at demonstrating the significance of the influence of the adhesive compositions ingredients mixing method on the strength properties of the tested epoxy compositions. Based on the obtained results, the following conclusions were presented:

- Mixing the components of the epoxy composition at higher speeds allows for higher tensile strength values, which may be due to better dispersion of the filler in the epoxy resin matrix.
- All modified compositions showed good interaction between the filler and the matrix in the form of epoxy resin, which is the basic assumption for the correct physical modification of the adhesive composition. A better distribution of the calcium carbonate filler at higher rotations at mixing can be noticed. Additionally, it can be seen from the SEM images that the wettability of the filler and the resin did not influence mixing speeds of the mixture, i.e., 460, 1170 and 2500 rpm.
- The highest tensile strength among modified compositions was obtained in the case of those containing the calcium carbonate filler. The compositions containing the active carbon filler showed the lowest tensile strength.
- In the mixing process, an important element, in addition to technological parameters, is also the method of mixing and the construction of the mixer, which affects both the degree of dispersion of the filler particles in the epoxy matrix, as well as the tensile strength of the filled epoxy adhesive composition.

As it has been observed, each of the analyzed factors has a significant impact on the quality of the performed tests and the effectiveness of the final results; therefore, the proper selection of technological factors is very important in the further bonding process.

The choice of the mixing method, type of equipment and technological parameters depends on many factors, including the type, properties and amount (and also proportions) of components, the form of components, the method of their preparation (also surface preparation) and the order of adding to be used for further applications. Therefore, it is extremely important to develop a technology for mixing the components of adhesive compositions, which will be the direction of further research.

## Figures and Tables

**Table 1:** The chemical formulas for the materials used in the tests.

Material Used in the Tests	Chemical Formula
Epidian 57 epoxy resin	${\mathrm{CH}}_{2}\begin{array}{c} \begin{array}{c} \;\\ - \end{array}\\ \backslash /\\ O \end{array}\mathrm{CH}-{\mathrm{CH}}_{2}-O$ ${\left[-C_{6}H_{4}-\begin{array}{c} \begin{array}{c} {\mathrm{CH}}_{3}\\ |\\ C \end{array}\\ |\\ {\mathrm{CH}}_{3} \end{array}-C_{6}H_{4}-O-{\mathrm{CH}}_{2}-\begin{array}{c} \;\\ \begin{array}{c} C\\ |\\ \mathrm{OH} \end{array} \end{array}H-{\mathrm{CH}}_{2}-O\right]}_{n}-C_{6}H_{4}-\begin{array}{c} \begin{array}{c} {\mathrm{CH}}_{3}\\ |\\ C \end{array}\\ |\\ {\mathrm{CH}}_{3} \end{array}-C_{6}H_{4}-O-{\mathrm{CH}}_{2}-\mathrm{CH}\begin{array}{c} \begin{array}{c} \;\\ - \end{array}\\ \backslash /\\ O \end{array}{\mathrm{CH}}_{2}$
Z-1 curring agent	$\begin{array}{r} H_{2}N-{\mathrm{CH}}_{2}-{\mathrm{CH}}_{2}-\mathrm{NH}-{\mathrm{CH}}_{2}-CH_{2}\\ |\\ {\mathrm{NH}}_{2}-{\mathrm{CH}}_{2}-{\mathrm{CH}}_{2}-\mathrm{NH} \end{array}$
NanoBent ZR2 montmorillonite	M_x_ [Al_4−x_Mg_j_] (Si_8_)O_20_(OH)_4_
Calcium carbonate	CaCO_3_
CWZ-22 active carbon	C

**Table 2:** Amount of ingredients and designation of adhesive compositions used in the tests.

Epoxy Resin	Curing Agent	Filler	Filler Quantity (in Ratio to the Weight of the Epoxy Resin)	Designation of the Epoxy Composition
Epidian 57 (100 g)	Z-1 (10 g)	-	-	E57/Z-1/100:10
		NanoBent ZR2 montmorillonit	5%	E57/Z-1/ZR2/100:10:5
		CaCO_3_ calcium carbonate	20%	E57/Z-1/CaCO_3_/100:10:20
		CWZ-22 active carbon	20%	E57/Z-1/CWZ-22/100:10:20

**Table 3:** Parameters of each mixing method.

Mixing Variant	Mixing Parameters				
	Blade Mixer (460 rpm, Time 3 min)	Dispersive Disc Mixer (460 rpm, Time 3 min)	Deaeration During the Mixing Process (3 min)	Deaeration After Complete Mixing (2 min)	Epoxy Resin Heating (to 50 °C)
W1	+	x	x	x	x
W2	x	+	x	x	x
W3	x	+	+	x	x
W4	x	+	+	+	x
W5	x	+	+	x	+
W6	x	+	+	+	+

**Table 4:** Parameters of each mixing variant.

Mixing Variant	Mixing Process Description
W1	Mixing with blade mixer at 460 rpm mixing speed in 3 min
W2	Mixing by dispersing disc mixer at 460 rpm mixing speed in 3 min
W3	Mixing by dispersing disc mixer with the speed of 460 rpm in 3 min, deaeration of the composition during mixing.
W4	Mixing by a dispersing disc mixer with the speed of 460 rpm in 3 min, deaeration of the composition during mixing and deaeration after the mixing process in 2 min
W5	Heating up the epoxy resin to 50 °C, mixing with a dispersing disc mixer at 460 rpm in 3 min, deaeration of the composition during mixing
W6	Heating up the epoxy resin to 50 °C, mixing with a dispersing disc mixer at 460 rpm in 3 min, deaeration of the composition during mixing and deaeration after the mixing process in 2 min

**Table 5:** Results of the Shapiro–Wilk tensile strength test of adhesive compositions grouped by mixing speed.

Adhesive Composition	Mixing Speed	Statistical Value *W* Shapiro–Wilk Test	Level *p* for the Shapiro Wilk Test
E57/Z-1/100:10	460 rpm	0.840221	0.165534
E57/Z-1/ZR2/100:10:5		0.888486	0.349546
E57/Z-1/CaCO_3_/100:10:20		0.979546	0.932177
E57/Z-1/CWZ-22/100:10:20		0.898619	0.402299
E57/Z-1/100:10	1170 rpm	0.797887	0.077876
E57/Z-1/ZR2/100:10:5		0.911598	0.477252
E57/Z-1/CaCO_3_/100:10:20		0.917350	0.512989
E57/Z-1/CWZ-22/100:10:20		0.952351	0.753973
E57/Z-1/100:10	2500 rpm	0.827686	0.133654
E57/Z-1/ZR2/100:10:5		0.834896	0.151300
E57/Z-1/CaCO_3_/100:10:20		0.950782	0.742774
E57/Z-1/CWZ-22/100:10:20		0.936241	0.639502

**Table 6:** Results of the Levene tensile strength test of adhesive compositions as a function of mixing speed.

Mixing Speed	Value of Levene *F* Test Statistics	Level *p* for the Levene Test
460 rpm	0.403671	0.752354
1170 rpm	5.485141	0.008722
2500 rpm	1.545882	0.241282

**Table 7:** Results of the Kruskal–Wallis test of multiple comparisons of average values of tensile strength of adhesive compositions grouped in relation to mixing speed.

Mixing Speed	Adhesive Composition	Average Tensile Strength (MPa)	The Value of the *R*-Rank Correlation Coefficient in the Kruskal–Wallis Test		
			460 rpm	1170 rpm	2500 rpm
460 obr/min	E57/Z-1/100:10	36.36		1.00	0.03
1170 obr/min	E57/Z-1/100:10	37.18	1.00		0.02
2500 obr/min	E57/Z-1/100:10	55.36	0.03	0.02	
460 obr/min	E57/Z-1/ZR2/100:10:5	14.21		0.23	0.00
1170 obr/min	E57/Z-1/ZR2/100:10:5	17.40	0.23		0.23
2500 obr/min	E57/Z-1/ZR2/100:10:5	22.96	0.00	0.23	
460 obr/min	E57/Z-1/CaCO_3_/100:10:20	29.03		0.23	0.02
1170 obr/min	E57/Z-1/CaCO_3_/100:10:20	32.24	0.23		1.00
2500 obr/min	E57/Z-1/CaCO_3_/100:10:20	35.04	0.02	1.00	
460 obr/min	E57/Z-1/CWZ-22/100:10:20	13.86		0.03	0.02
1170 obr/min	E57/Z-1/CWZ-22/100:10:20	18.58	0.03		1.00
2500 obr/min	E57/Z-1/CWZ-22/100:10:20	18.94	0.02	1.00	

## Data Availability

Data is contained within the article.

## Appendix A

**Table A1:** Results of the Shapiro–Wilk tensile strength test of adhesive compositions.

Adhesive Composition	Mixing Variant	Statistical Value *W* Shapiro-Wilk Test	Level *p* for the Shapiro Wilk Test
E57/Z-1/100:10	W1	0.931608	0.607380
E57/Z-1/ZR2/100:10:5		0.780202	0.055341
E57/Z-1/CaCO_3_/100:10:20		0.921542	0.539937
E57/Z-1/CWZ-22/100:10:20		0.793543	0.071712
E57/Z-1/100:10	W2	0.982155	0.945810
E57/Z-1/ZR2/100:10:5		0.955317	0.775054
E57/Z-1/CaCO_3_/100:10:20		0.952447	0.754651
E57/Z-1/CWZ-22/100:10:20		0.924226	0.557563
E57/Z-1/100:10	W3	0.883807	0.326920
E57/Z-1/ZR2/100:10:5		0.853655	0.206350
E57/Z-1/CaCO_3_/100:10:20		0.953451	0.761801
E57/Z-1/CWZ-22/100:10:20		0.937770	0.650218
E57/Z-1/100:10	W4	0.956981	0.786817
E57/Z-1/ZR2/100:10:5		0.939913	0.665321
E57/Z-1/CaCO_3_/100:10:20		0.921252	0.538048
E57/Z-1/CWZ-22/100:10:20		0.889458	0.354383
E57/Z-1/100:10	W5	0.840221	0.165534
E57/Z-1/ZR2/100:10:5		0.888486	0.349546
E57/Z-1/CaCO_3_/100:10:20		0.979546	0.932177
E57/Z-1/CWZ-22/100:10:20		0.898619	0.402299
E57/Z-1/100:10	W6	0.838717	0.161408
E57/Z-1/ZR2/100:10:5		0.994039	0.991752
E57/Z-1/CaCO_3_/100:10:20		0.841071	0.167903
E57/Z-1/CWZ-22/100:10:20		0.884104	0.328323

**Table A2:** Results of Levene test of tensile strength of adhesive compositions in grouping into mixing methods.

Mixing Variant	Value of Levene *F* Test Statistics	Level *p* for the Levene Test
W1	2.177346	0.130524
W2	1.811443	0.185664
W3	1.479973	0.257679
W4	1.876855	0.174194
W5	0.403671	0.752354
W6	2.567455	0.090737

**Table A3:** Results of Tukey’s post hoc (HSD) tensile strength test for E57/Z-1/100:10 composition.

Mixing Variant	Average Tensile Strength (MPa)	Homogenous Groups			
		1	2	3	4
W1	16.93	***			
W2	18.97	***			
W3	27.90		***		
W4	30.08		***	***	
W5	36.36			***	***
W6	38.93				***

**Table A4:** Results of Tukey’s post hoc (HSD) tensile strength test for E57/Z-1/ZR2/100:10:5 composition.

Mixing Variant	Average Tensile Strength (MPa)	Homogenous Groups	
		1	2
W1	12.54	***	
W2	13.06	***	
W3	14.27	***	***
W4	14.63	***	***
W5	14.21	***	***
W6	16.32		***

**Table A5:** Results of Tukey’s post hoc (HSD) tensile strength test for E57/Z-1/CaCO_3_/100:10:20 composition.

Mixing Variant	Average Tensile Strength (MPa)	Homogenous Groups			
		1	2	3	4
W1	13.39				***
W2	20.65	***			
W3	23.64	***			
W4	24.59	***	***		
W5	29.03		***	***	
W6	31.50			***	

**Table A6:** Results of Tukey’s post hoc (HSD) tensile strength test for E57/Z-1/CWZ-22/100:10:20 composition.

Mixing Variant	Average Tensile Strength (MPa)	Homogenous Groups		
		1	2	3
W1	7.42			***
W2	10.19		***	***
W3	12.94	***	***	
W4	13.56	***	***	
W5	13.86	***		
W6	15.94	***		
